# Kaupapa Māori development of te reo Māori assessments and culturally responsive hearing healthcare in Te Waipounamu: findings from wānanga

**DOI:** 10.1080/03036758.2024.2418999

**Published:** 2024-10-30

**Authors:** Alehandrea Raiha Manuel, Jennifer Smith, Tare Lowe, Greg A. O’Beirne

**Affiliations:** a Faculty of Medical and Health Sciences, School of Population Health, Waipapa Taumata Rau | The University of Auckland, Aotearoa, New Zealand; b Eisdell Moore Centre, Waipapa Taumata Rau | The University of Auckland, Aotearoa, New Zealand; c Child Well-being Research Institute, Te Whare Wānanga o Waitaha | The University of Canterbury, Aotearoa, New Zealand; d School of Psychology, Speech and Hearing, Te Whare Wānanga o Waitaha | The University of Canterbury, Aotearoa, New Zealand

**Keywords:** Te Reo Māori, Indigenous languages, audiology, assessments, healthcare, hearing health, Māori health, Kaupapa Māori

## Abstract

The development of valid and robust te reo Māori (te reo) hearing assessments in Aotearoa New Zealand (Aotearoa) is an area of research that requires further attention from a Kaupapa Māori stance. In support of te reo revitalisation, this article shares findings from two wānanga in Te Waipounamu (the South Island of Aotearoa) with research partners (hearing healthcare professionals and Māori health and education professionals). Research partners’ perspectives and experiences of barriers and facilitators to ear and hearing healthcare in Te Waipounamu, development of te reo assessments, and their application in clinical and community settings are explored. Embedded within Kaupapa Māori theory and methodology, data was analysed using reflexive thematic analysis to identify commonalities and patterns in ‘Hauora Māori Community’ and ‘Hearing Healthcare Community’. This paper presents seven whakaaro themes and first steps from provider perspectives towards the development of culturally appropriate and responsive hearing healthcare tools and services.

## Abstract (es)

**Glossary of Māori words:** Ahitereiria ki te Hauāuru: Western Australia; Āhuatanga Māori: All things Māori; Aotearoa: North Island of New Zealand – now used as the Māori word for New Zealand; Aroha: Compassion; Atua kaitiaki Māori: Māori ancestor/guardian with continuing influence; Awhi: Support; deaf: With a lowercase ‘d’, deaf refers to hearing loss – e.g. ‘he is deaf’; Deaf: The word Deaf (with a capital ‘D’) denotes a unique community. The use of sign language as one’s first language is the principle characteristic of people who identify with this community; Hauora Māori: Māori health and wellbeing; Hapori Māori: Māori community; He Awa Whiria: Braided rivers approach; ia: Te reo Māori pronoun for everyone; Iwi: Tribe; Kai: Food; Kāi Tahu/Ngāi Tahu: Tribal group of the South Island; Kaitiaki: Guardian and caregiver; Karakia: Incantation, chant, thought, or prayer; Karakia Tīmatanga: Opening thought/prayer; Karakia Whakamūtunga: To close the wānanga; Kaumātua: Māori elder/s; Kaupapa: Topic/policy/matter of discussion; Kaupapa Māori: By Māori, for Māori, with Māori philosophical approach using Māori knowledge and values; Koha: A gift/custom of gifting; Kōrero: Narrative/s and prose; Kūmara: Sweet potato; Mana: Power; Mana-enhancing: Mutual respect and commitment to caring for each other’s mana; Manaaki: Ethic of care and respect; Māori: Indigenous people of Aotearoa New Zealand; Mātauranga Māori: Indigenous Māori knowledge; Mihi whakatau: Formal welcome, speech of greeting; Mihimihi: Greetings and acknowledgements; NZSL: Deaf New Zealand Sign Language; Ngāpuhi: A tribal group situated in the top of the North Island; Ngāti Whātua: Tribal group between the Hokianga Harbour and Tāmaki; Ōtautahi: Traditional name known now as Christchurch; Pākehā: European New Zealander, non-Māori; Pākeke: Adults; Rangatira: Leaders; Rōpū: Groups; Rūnanga Māori: Māori board/council of governance; Tamariki: Child/ren; Tamariki reo Māori: Children who speak Māori; Tangata Tiriti: People of te Tiriti, referring to all non-Māori citizens and residents of Aotearoa; Tangata Whenua: People of the land, Indigenous peoples, local people, hosts; Taonga: Treasure, things of social or cultural value; Tauiwi: foreign people, non-Māori; Te Araroa: A rural town along the East Cape of the North Island; Te hitori: The history; Te reo Māori (te reo): The Māori language, Indigenous language of Aotearoa; Te Tairāwhiti: lit. ‘the coast upon which the sun shines across the water’, an East Coast region of the North Island of Aotearoa; Te Tai Tokerau: lit. the North Coast, Northland; Te Tiriti o Waitangi (Te Tiriti): Signed in 1840 this is the Māori language version of an agreement between two internationally recognised sovereign nations, Māori, as Tangata Whenua, and the British Crown; Te Waipounamu: The South Island of Aotearoa; Te Whakamātautau Whakarongo o Aotearoa: Name of a te reo Māori digit triplet test, TRMDTT; Tēnei rangahau: This research; Tikanga: Ethics, customs, practices, protocols, system of values; Tino Rangatiratanga/Rangatiratanga: Absolute sovereignty, self-determination; Tuakana-Teina: Older-younger; Turi Māori: Deaf Māori; Wānanga: Traditional method for knowledge sharing; Wāwata: Aspirations; Whakaaro: ways of thinking/thoughts/considerations or cast attention to; Whakakapi: Summary, conclusions, reflections; Whakamā: Fear and shame from services; Whakapapa: To place in layers, to recite genealogies, lineage; Whakawhanaungatanga: An indigenous process of creating relational connection; Whānau Ora: A culturally-based and whānau-centred approach; Whānau: Immediate and extended family and friends; Whenua: Land; Whetūrangitia: lit. stars that adorn the sky or the transformation of loved ones who have passed away, transcending from their earthly bodies to become stars.

## Mihi whakatau – welcome

*Tukua te wairua kia rere ki ngā taumata. Hai ārahi i ā tātou mahi. Me tā tātou whai i*

*ngā tikanga a rātou mā. Kia mau kia ita. Kia kore ai e ngaro. Kia pupuri.*

*         Kia whakamaua. Kia tina. Tina. Hui e. Tāiki e*.

Kei te mihi ki ngā ātua kaitiaki o te ao Māori, nā koutou i tiaki ngā mea katoa.

Kei te mihi atu ki a rātou kua whetūrangitia, haere, haere, haere atu rā.

Kei te mihi mātou (AM, JS, TL, GO) ki a koutou (communities, experts, scholars, research partners and wider EMC project team). We acknowledge your efforts in the revitalisation of te reo Māori (te reo), the indigenous Māori language of Aotearoa New Zealand (Aotearoa).

Nō reira, tēnā koutou, tēnā koutou, tēnā koutou katoa.

We open this article with a karakia tīmatanga (opening thought/prayer), ‘Tukua te wairua’, and a mihimihi (greetings and acknowledgements) to acknowledge the guardians and ancestors of the Māori (Indigenous peoples of Aotearoa) world, including the stars that adorn the sky, all who have guided this kaupapa (topic/policy/matter of discussion), and each person who reads this article in the pursuit to preserve Māori traditions including te reo. This article is modelled on the processes of each wānanga (traditional method for knowledge sharing) in this project. This includes karakia (incantation, chant, thought, or prayer), mihimihi and whakawhanaungatanga (an indigenous process of creating relational connection), an introduction to te hitori (the history) and kaupapa, tikanga (ethics, customs, practices, protocols, system of values), kai (food), collective and individual whakaaro (thoughts, opinions, and considerations), and whakakapi (summary, conclusions, reflections).

As part of whakawhanaungatanga, we introduce ourselves within this paper and our shared commitment towards growing research that is responsive to Māori. Our collective efforts are part of a te reo revitalisation project. This comes with our responsibility to enact Te Tiriti o Waitangi (Te Tiriti) and provide accessible, equitable, intergenerational, transformative ear and hearing healthcare that is by Māori, for Māori. We are:

Alehandrea (she/her/ia) [AM]: Nō Te Araroa, Te Tairāwhiti tōku pāpā. Nō Philippines (Ilocos) tōku māmā. My work in this kaupapa, is inspired by the generations of rangatira (leaders) and communities asserting tino rangatiratanga (absolute sovereignty, self-determination) and reclaiming their rights to te reo. I have enjoyed learning from colleagues, the wider project team, and the communities we have worked alongside with the shared commitment to protect te reo and mātauranga Māori (Indigenous Māori knowledge) for generations to come.

Jennifer [JS]: Nō te whenua o Te Tai Tokerau tēnei kūmara. Nō Ngāti Whātua, nō Ngāpuhi tōku whakapapa. I am hard of hearing and a second language speaker of te reo. Just like my ancestors, colleagues and loved ones, I strive in service of our past, present, and future generations. I am consistently humbled by the beautiful people, communities and researchers alike, who are committed to this kaupapa; mei kore ake koutou hei ārahi i a au, ānei ōku mihi maioha.

Tare [TL]: Ko Kāi Tahu te iwi. I am a Māori audiologist and emerging Kaupapa Māori researcher. I feel immense privilege to have had the opportunity to contribute to this kaupapa. I learnt so much from the communities in working towards equitable hearing healthcare and I will be forever grateful for their time and contributions.

Greg [GO]: Ko Tauiwi te iwi. He Pākehā au nō Ahitereiria ki te Hauāuru. As Tangata Tīriti (a person of Te Tiriti), I am committed to striving to reduce inequities in hearing health outcomes and want to thank our partners in both these wānanga for sharing their perspectives.

## Te Kaupapa

### Te Hitori (the history)

In Aotearoa, Te Tiriti o Waitangi (Te Tiriti) – a treaty agreement between the British Crown and Māori (the Indigenous peoples of Aotearoa) – was signed by over 500 Māori rangatira (leaders) in 1840. In Te Tiriti (which, under the legal doctrine of *contra proferentem*, has precedence over the English version of the treaty), the British Crown promised Tangata Whenua (people of the land) the rights and privileges of British subjects and affirmed Māori rangatiratanga over their affairs, including natural resources (Te Puni 2001; Orange 2011; Came et al. 2020).

Immediately after Te Tiriti was signed, policies of assimilation were put in place by the British Colonial Government, with the intent to absorb Māori into colonial culture (Ka'ai-Mahuta 2011). Implementation of the terms of Te Tiriti is continuously resisted by colonial leadership, with colonial hegemony and settler interests being favoured at the expense of Māori sovereignty – this was especially evident in the State education system (Moewaka Barnes and McCreanor 2019). Consequently, Māori have been denied their rights to their whenua (land), kinship connections, language, culture, and equitable health outcomes.

The profound impacts of colonisation and assimilation span across generations of Māori, including hard-of-hearing and Turi (Deaf) Māori (Smiler 2014; Manuel 2022). For example, the suppression of te reo and sign language has had a significant impact on Māori cultural and political identity. As Ka'ai-Mahuta (2011) commented, ‘although the Māori community has never forfeited its mana [power] or denied its cultural uniqueness, the policies of monoculturalism continually place it under stress’ (p. 218).

Since the 1980s, there has been a series of Kaupapa Māori (‘by Māori, for Māori, with Māori’ philosophical approach using Māori knowledge and values) education strategies, developed as resistance initiatives outside the Western education system (Smith 2003; Walker 2016). This has also translated to healthcare, with an influx of Kaupapa Māori healthcare initiatives (Rolleston et al. 2020). In hearing healthcare there are limited discussions on supporting cultural values and the views of those in most need. Moreover, there is a scarcity of hearing healthcare research, initiatives, assessments and tools from a Kaupapa Māori stance. This is despite disparities in hearing healthcare and outcomes between Māori and non-Māori (Manuel 2022).

### A right to language

Under Article 13(1) of the United Nations Declaration on the Rights of Indigenous Peoples (UNDRIP), Indigenous peoples have the right to revitalise, use, develop and transmit to future generations their languages, oral traditions, histories, philosophies, writing systems and literature (G.A. Res. 61/295 2007). In Aotearoa, language is vital to Māori hard-of-hearing and d/Deaf people identity/ties and is a channel for empowerment and rangatiratanga (Smiler 2006; Smiler and McKee 2007; Lowe 2023). Hard-of-hearing kaumātua (Māori elder/s) and parents of deaf tamariki Māori (Māori children) have expressed the importance of te reo and its role in helping future generations to learn tikanga Māori and communicate with te reo speakers (Crisp 2010; Manuel 2022). Dr Moana Jackson (1987) stated that the terms of Te Tiriti protected Māori culture, institutions, and ways of life, and that ‘they were mechanisms to protect their taonga [treasure, things of social or cultural value]’ including te reo (pg.48). Parallel to Jackson’s kōrero (narrative and prose), Lowe (2023) and Dawson (2022) emphasise the importance of Te Tiriti, community partnerships, and the critical need for developing and implementing te reo assessments in audiology, such as ‘Te Whakamātautau Whakarongo o Aotearoa’ – a te reo Digit Triplet Test (TRMDTT).

### Tēnei rangahau

Tēnei rangahau (this research) is part of a wider project in Te Waipounamu (the South Island of Aotearoa) that is focused on researchers and clinicians developing connections and partnerships with rūnanga Māori (Māori board/council of governance) and communities to create and develop adult te reo hearing assessments through a Kaupapa Māori approach. It follows from studies looking into TRMDTT, first developed by Christa Murray (Kāi Tahu) and Alice Bowden under the supervision of Greg O’Beirne and Jeanette King at the University of Canterbury (Murray 2012; Bowden 2013). Dawson (2022) continued the validation process and explored the development of te reo audiological assessment tools through culturally responsive ‘He Awa Whiria’ (braided rivers) and ‘Community-Up’ approaches. Subsequently, Lowe (2023) investigated the lived experiences, perspectives and attitudes towards these te reo tools and hearing healthcare in Te Waipounamu. Further exploration of Māori and non-Māori provider perspectives of hearing healthcare was a key recommendation from Lowe’s study. Findings from these studies are detailed in 2024 papers (Dawson et al. 2024 and Lowe et al. 2024).

This research focused on exploring and understanding the experiences and perspectives of the ‘Hauora Māori Community’ (HMC) and ‘Hearing Healthcare Community’ (HHcC) on barriers and facilitators to ear and hearing healthcare, development of te reo hearing assessments and their application in Te Waipounamu. In this article, we bring forward their narratives and present future directions that have stemmed from discussions in wānanga.

### Kaupapa Māori and He Awa Whiria research approaches

This research is underpinned by Kaupapa Māori and He Awa Whiria approaches to research. Kaupapa Māori research (KMR) stemmed in response to the marginalisation of Māori communities perpetuated by the ‘othering’ and deficit-focused approaches in social science research (Smith 1999). Māori are placed at the centre of enquiry in KMR where Māori realities are seen as legitimate, power dynamics are challenged and social transformation within systems can occur (Smith 2012; Tōmaiora Māori Health Research Group 2015). The following KMR principles guided this research: careful consideration of research partnerships; Māori have control over the construction, analysis and interpretation of Māori data; organise and interpret findings that hold value for Māori communities and are informed by mātauranga Māori and Māori worldviews; be aligned with a ‘structural determinants’ approach that critiques issues of power and deficit framing and promotes transformation and social justice; be supportive of decolonisation and accepting of diverse Māori realities; and contribute towards future layers of Kaupapa Māori theory and practice (Smith 1999; Walker et al. 2006; Smith 2015; Curtis 2016).

This research is further grounded by ‘He Awa Whiria’, the innovative braided rivers framework designed by Macfarlane and Macfarlane (2019). He Awa Whiria draws inspiration from Indigenous and Western streams of knowledge, while maintaining a consciousness of Māori sovereignty. Dawson and colleagues (2024) present a He Awa Whiria framework within an audiological research and hearing healthcare model that facilitates the integration of Māori knowledge and cultural values. In adapting these approaches, a culturally safe strengths-based space was created within wānanga so the ‘Hauora Māori Community’ (HMC) and ‘Hearing Healthcare Community’ (HHcC) could express their unique perspectives without prejudice. The research team (AM, JS, TL, GO) could also focus on the strengths inherent in both communities.

### Ethics approval

The study was approved by The University of Canterbury Human Research Committee (ref.no. HREC 2022/09/LR). Consultation with iwi (tribe) through the Ngāi Tahu Consultation and Engagement Group at the University of Canterbury occurred and was approved in March 2022.

### Wānanga

Narratives were shared through a process of wānanga. The evolution of wānanga as a traditional indigenous method centres on knowledge construction and transmission. Mahuika and Mahuika (2020) discuss the growing use of wānanga in Māori research practice. It takes different forms and practices but was chosen here for two reasons. Firstly, it is a preferred method for knowledge sharing when researching āhuatanga Māori (all things Māori). Secondly, it provides a framework of tikanga Māori, preferred by the Māori researchers, as a guide to hosting others in culturally safe ways (Mead 2003).

Two wānanga, one with the Hauora Māori Community (HMC) and one with the Hearing Healthcare Community (HHcC), were facilitated by the research team in 2022 at the University of Canterbury Rehua building in Ōtautahi (Christchurch). Research partners in these communities had the option of attending face-to-face or online via Zoom. For HMC wānanga, we invited Māori (hearing, d/Deaf, hard-of-hearing) providers with an interest in ear and hearing healthcare. Education and health professionals in hearing healthcare were invited for the HHcC wānanga. If a person was eligible for both wānanga they were given a choice of which they attended.

The wānanga process (outlined in ‘Mihi Whakatau’ section) allowed for a comprehensive understanding of the historical and contemporary experiences of Māori in the realm of hearing healthcare and enabled research partners to think openly and outside their organisation roles, engaging in active and collective thinking and problem-solving. HMC and HHcC wānanga schedules were used as a guide, and formulated according to the research aim, objectives, and prior studies by Dawson (2022) and Lowe (2023) for each community. In both wānanga, research partners were asked to reflect on Māori health training, practices with Māori patients/clients and whānau (immediate and extended family and friends), how hearing healthcare could be mana-enhancing (mutual respect and commitment to caring for each other’s mana), pros/cons and pragmatic integration of te reo, and what support is needed to provide a culturally safe and responsive practice.

The wānanga were audio-recorded through the ‘Zoom’ video conferencing platform, transcribed using commercial transcription software, and transcripts were checked for accuracy by AM and TL. HMC and HHcC research partners were provided with a summary of their quotes, opportunities to discuss their quotes and, if preferred, to select how those quotes were to be attributed (either their own name, a pseudonym of their choice, or a randomly generated pseudonym). These attributions appear in parentheses after each quote below.

Seventeen research partners contributed to the wānanga (see Table 1). Two qualified NZSL (Deaf New Zealand Sign Language) interpreters translated between NZSL, te reo Māori, and English.

**Table 1.** Research partners from each wānanga.

Hauora Māori Community: HMC	Hearing Healthcare Community: HHcC
1. Christina 2. Jeanine 3. Irihāpeti 4. Marion 5. Kiri 6. Kaiwhakapuawai 7. Jen	1. Tracey 2. Brandt 3. Beth 4. Katie 5. Jillian 6. Rosie 7. Debbie 8. Madeleine 9. Naomi 10. Ellie

The HHcC included: educators for the Deaf, audiologists, an audiometrist, a Vision and Hearing technician, a hearing therapist and speech-language therapist, and clinical educators. Hearing, hard-of-hearing and d/Deaf were part of this community. Two worked for a non-government organisation, one identified as Māori. The HMC included: Māori health promoters, kaitiaki (guardian and caregiver), a community health worker, and academics. We acknowledge the intersectionalities of the Māori research partners in this study as community members with lived experiences of hearing loss, being hard-of-hearing or d/Deaf, and as support for whānau members who have had ear and hearing difficulties.

### Reflexive thematic analysis

Transcribed data was input into NVivo 12 software to analyse and organise data gathered from each wānanga. A six-phase reflexive thematic analysis (RTA) recursive process (Braun and Clarke 2021) served as a tool to assist in the inductive identification of commonalities and patterns of meaning across the wānanga data while being reflexive of KMR and our own positionalities, worldviews, and biases. The research team independently read through both transcriptions. AM put together the initial coding framework, with JS, TL, and GO providing feedback on codes. Several discussions and iterations of wānanga themes and sub-themes followed in team meetings, with Māori researchers (AM, JS, TL) ensuring the analysis and interpretation of data appropriately aligned with KMR principles.

Koha, a common tikanga (protocol/custom) in Māori tradition, is part of forming reciprocal relationships (Mead 2003). It is often recognised as the cultural protocol of gifting to recognise and affirm the mana (power) of both the giver and recipient. Koha takes different shapes and forms, including that of whakaaro (ways of thinking/thoughts/considerations or to cast attention to). Perrett (2003) claims ngā whakaaro Māori or Māori ways of thinking, are part of rich knowledge baskets that are living and still growing in intellectual tradition. Mika and Southey (2018) argue that within a Māori worldview, a whakaaro method including that of analysis actively stimulates Māori imagination and fosters creative thinking and theoretical practice.

In acknowledgement of this kōrero, as well as in alignment with Kaupapa Māori and He Awa Whiria research praxis and principles, the analysis of data should respect the meaning and understanding of whakaaro from each wānanga. In light of this, the research team recognised that further condensing and merging of whakaaro themes, subthemes, and codes in the thematic analysis process would potentially diminish the whakaaro and therefore, the mana of our hapori Māori (Māori community) research partners. The whakaaro shared in the next section embody KMR principles, capture the voices of both Māori and non-Māori partners, and present a coherent narrative of realities in the hearing healthcare sector.

## Whakaaro

This section is named ‘Whakaaro’ to honour the seventeen research partners’ mana and their fluid and evolving intellectual discourse from each wānanga. The research partners’ contributions of whakaaro cannot be understated; during the wānanga, they cast our attention to their stories and experiences, their emotions, their feelings and their wāwata (aspirations) for the sector.

**Figure 1.**
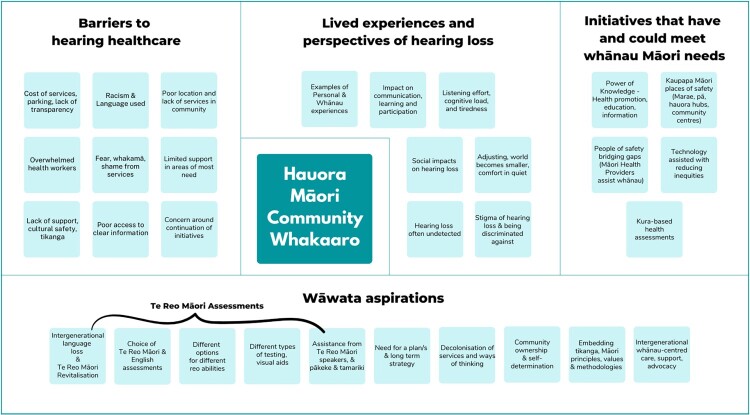
Hauora Māori Community (HMC) Whakaaro.

**Figure 2.**
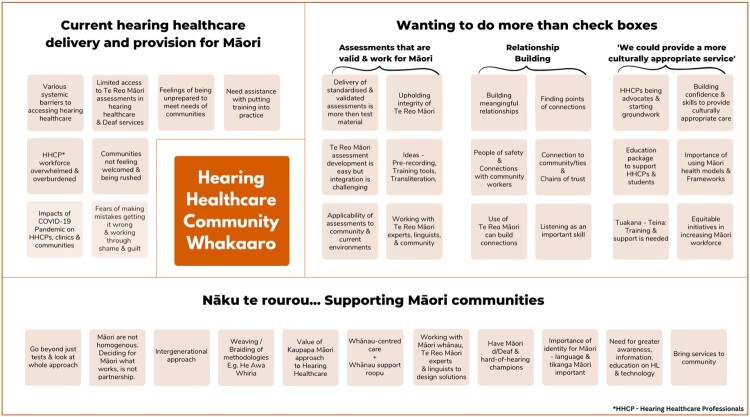
Hearing Healthcare Community (HHcC) Whakaaro.

Figures 1 and 2 illustrate the theming of whakaaro within each wānanga. A summary of whakaaro themes and selected sub-themes from each wānanga is shared below. A comprehensive report detailing each theme discussed in the wānanga, will be made available in time.

### Wānanga – Hauora Māori Community (HMC) whakaaro

Four whakaaro themes were identified within the HMC wānanga (see Figure 1): (1) barriers to hearing healthcare; (2) lived experiences of hearing loss; (3) initiatives that have and could meet whānau Māori needs; (4) wāwata (aspirations).

#### Barriers to hearing healthcare

Several barriers to hearing healthcare were discussed within the wānanga, including feeling unsafe, bias and discrimination, whakamā (fear and shame) from services, cost and lack of transparency, location, lack of support and poor access to information. A definition of ‘feeling unsafe’ was not stated, however, HMC gave examples of their own and community experiences of feeling afraid to attend hearing healthcare.

You know pākeke [adults] are afraid to go to the hospital. Let alone go to our audiologist. (Kaiwhakapuawai)

Several research partners reported that some of this may stem from experiences of biased healthcare professionals, such as preconceptions and assumptions that tamariki reo Māori (children who speak Māori) do not understand what is going on. The HMC further reported limited access to information on hearing loss and prevention, technology funding, hearing health and support programmes, services, and support. The lack of available information impacted community awareness of existing hearing health services, funding, and programmes in their communities.

#### Lived experiences and perspectives of hearing loss

The HMC stated that Māori experiences of middle-ear issues and hearing loss are not homogenous. Several HMCs reported personal and whānau experiences of the impacts of hearing loss on cognition, tiredness, and the ability to participate and get involved. For example, Jeanine spoke about the impacts of middle ear issues on her son’s balance and ability to participate in sport in the way he would have liked.

Stigma of hearing loss was frequently brought up by research partners. Several reported being discriminated against for having hearing loss and wearing hearing technology.

I got told my career would have to change and I was like, but I didn't want to ever be anything other than a teacher. (Jen)

Whakamā was shared in the sense of feeling ashamed, embarrassed, or being worried about the implications of hearing loss, all of which could impact decisions to access hearing healthcare.

#### Initiatives that have and could meet whānau Māori needs

A common thread amongst the HMC was that health promotion and education regarding ear and hearing health is key. This includes techniques similar to ‘blow breathe cough’ (Barker and Thomas 1994).

This nose-blowing technique has changed my life for her [Irihapeti’s daughter], because we didn't have to go through surgery, we didn't have to do anything invasive, and she was like “oh choice”. (Irihapeti)

All research partners discussed the importance of safe places and people to build confidence and trust in hearing healthcare across generations. Those who worked in the community highlighted whānau ora (a culturally-based and whānau-centred approach) service and navigator roles in assisting whānau through hearing healthcare, such as filling out forms/applications, and supporting whānau through services.

It's about reorientation of services to the marae and I guess you know that's a safe place to be for whānau. (Christina)

The use of technology and community-based healthcare developed with the community, hearing assessments at schools and marae, and good communication were identified as important in creating a safe healthcare environment. Several reported experiencing poor communication between ear and hearing healthcare services and difficulties with accessing their information from healthcare providers, thus impacting the continuation of care. Once whānau felt listened to and their needs were met, there were generally positive experiences.

I was just fortunate to actually get a decent GP [General Practitioner] that actually took me through that, and I got seen [by an Ear, Nose, Throat specialist] relatively quickly. (Kiri)

#### Wāwata (aspirations)

Through research partners’ kōrero, it was clear that there was more to the delivery of standardised and validated assessments than simply the test material. A choice of a te reo or English assessment was seen as a step forward, especially for generations to come. Te reo assessments were viewed as a medium within healthcare to revitalise te reo, which is an important part of many Māori identity/ties. Ideas were put forward for future te reo hearing assessments, like the inclusion of picture-pointing tasks and the use of recorded stimuli to ensure correct pronunciation.

There were concerns among research partners around ensuring the continuation of initiatives. Several HMCs highlighted the need for long-term thinking in strategic planning that builds on what has already been demonstrated to improve care and outcomes for Māori.

It's not reinventing or making a new wheel, it's seeing what worked for our kaumātua, and what works now for our tamariki [children] in this new area of technology and bringing it all on board. (Irihapeti)

Wāwata for current and future generations were shared at the table, including improved education on Aotearoa history and Te Tiriti, strengthening of whānau-centred care and community autonomy, and approaches based on shared values.

In the creation and sustainability of hearing healthcare interventions and policies, HMC research partners stated that whānau Māori and community autonomy and self-determination are imperative. In Jeanine’s words:

We've got to meet in the middle and not just rescue, we've got to educate and bring our whānau on a journey. So that they can then be empowered to push forward the process but it's the initiation of this process that is really key to have a full wrap-around of support and services. (Jeanine)

Irihapeti discussed starting with educating Tangata Tiriti about decolonisation of services and ways of thinking, and sharing ‘decolonisation Tiriti stories’, shifting away from stereotypical and deficit notions of Māori. Several of the HMC recognised this was often overlooked and undervalued in training ear and hearing health professionals, and that training and praxis should take on approaches based on shared values.

I just think come back to what makes us human and connects us as values … It's connection to others, it's relationships with others that helps you to advance whatever is the kaupapa you're carrying. (Jeanine)

### Hearing Healthcare Community (HHcC) Whakaaro

Conversations in the HHcC wānanga were centred around three whakaaro themes (see Figure 2): (1) current hearing healthcare delivery and provision; (2) wanting to do more than check boxes; (3) Nāku te rourou … Supporting Māori communities.

#### Current hearing healthcare delivery and provision

Barriers to accessing quality hearing healthcare were observed by the HHcC. For example, a majority of the HHcC discussed unfriendly hearing healthcare environments as factors impacting access to care. Descriptions of unfriendliness were based around communities not feeling welcomed, experiences of racism, lack of support and time from health professionals, feeling rushed within appointments and the appearance of clinics. Staff shortages and lower engagement with whānau Māori were also noted to be heightened since the COVID-19 pandemic.

When I do go out to the marae a lot of what I hear is ‘the audiologist just gave me these because they were cheap and nasty, and they wanted me out the door’. (Debbie W)

Several HHCPs felt unprepared to meet the needs of underrepresented communities, especially Māori communities. Consequently, there were fears of ‘getting it wrong’, ‘making mistakes’, and being judged not just by Māori communities but also by their own hearing healthcare professional community. This was regardless of whether they have or have not had training in the past.

I’m glad that I’m here [at this wānanga], but I could easily have not been and I would have just slipped through the crack. (Katie)

Many claimed there was a lack of financial and non-financial support (including time flexibility) from workplaces to develop their culturally responsive care skills, requiring the clinicians to organise and fund this themselves.

You know that's all they talk about is the inequity and how it's important, but yeah they won't give the money for us to make it a training, to train the team, and it should be a big part of it. (Tracey)

#### Wanting to do more than check boxes

An unmet need reported by the HHcC was the limited access to te reo assessments in audiology, B4 school, and Deaf services. There was a consensus among the HHcC that a te reo assessment would open up options for more people. More specifically, HHcC supports the delivery of standardised and validated assessments that are replicable and applicable in various environments. They offered several ideas such as pre-recording assessments to assist in delivering replicable assessments.

The recording is actually a training for the deliverer of the test, and they can hear how it should be said, and then learn how to do it, properly. (Madeleine)

Ensuring that these new assessments were integrated into clinical practice in an authentic way, rather than a ‘checking the box’ exercise, would require additional training and support (described below). Debbie W, Rosie, Brandt and Katie discussed the important roles of clinicians in driving this change, developing the confidence to extend this aspect of their practice and provide culturally appropriate and responsive care in the long-term.

I think it was for me feeling comfortable or learning to feel comfortable in the discomfort of innovation. (Rosie)

Research partners brought up the need for training in holistic Māori approaches to care, learning about tikanga Māori and Māori values, and bringing ‘welcoming’ services to the community. Education packages and professional development activities that incorporate Māori health models and frameworks, tikanga Māori, and cultural safety that support practitioners and students were recommended. Within these conversations, were thoughts around braiding the best of Te Ao Māori and Western practices and values.

We now start with mihimihi and staff introduce themselves as people rather than as their roles … (Jillian)

The use of te reo and the ability to listen were identified as useful skills to start ‘making those links’, finding points of connections, and maintaining trust with Māori communities.

The biggest thing I find about that is then trying to get them to go to an audiologist from having seen us because they want, they like that connection. (Debbie W)

Those who have received base-level training required further support to put into practice. Tuakana-teina (older-younger) training support methods were suggested to alleviate feelings of being overwhelmed and overburdened.

Research partners saw increasing the Māori hearing healthcare workforce as a way of improving the delivery and provision of care, especially given the HHcC observations of Māori community health workers as people of safety and connectors to community/ties. Addressing the poor representation of Tangata Whenua in audiology was seen as a priority, as well as the need for equitable initiatives and pipeline approaches within training programmes to achieve this.

#### Nāku te rourou … Supporting Māori communities

The HHcC reported they want and need to support Māori communities. They recognised individual practitioner change is necessary but that they too experience systemic barriers.

How do we make it meaningful like to NZAS [New Zealand Audiological Society] because I thought it could be good if you get your annual practising certificate that you should have to demonstrate some sort of cultural safety in order to get that … How do we get through these barriers in the organisations that are pretty influential? (Beth)

They suggested: a need for greater awareness, information, and education on hearing loss and available hearing technology for Māori communities; an increase in whānau-centred care and whānau support rōpū (groups); and the importance of having hard-of-hearing and d/Deaf champions. In light of these suggestions, the HHcC recognised that Māori are not homogenous, particularly where hard-of-hearing and d/Deaf Māori have different intersectional experiences. They stated that deciding for Māori what works is not partnership and reported the need to work with whānau Māori. This too crossed over to te reo assessments. Research partners acknowledged working with te reo experts, linguists and Māori communities as imperative for upholding the integrity of te reo.

How do we know what works when we don't actually ask? (Katie)

## Whakakapi – summary and recommendations

*Language is the lifeline and sustenance of a culture. It provides the tentacles that*

*can enable a child to link up with everything in his or her world. It is one of the most*

*      important forms of empowerment that a child can have.*

e kī a Dr Rangimarie Turuki Arikirangi Rose Pere:

Educationalist, te reo advocate and academic (1997, p. 9)

There have been limited discussions with the health and education sectors around supporting Māori cultural values and te reo in hearing healthcare (EMC Working Group 2021; Manuel 2022; Lowe 2023; Wai Rangahau 2023). This study was designed with that in mind, to showcase the establishment and growing of partnerships between HMC, HHcC, and researchers. In this study, several barriers and facilitators to ear and hearing healthcare, development of te reo hearing assessments and their application in clinical and community settings in Te Waipounamu were brought forward by HMC and HHcC. Kaupapa Māori and He Awa Whiria approaches created a platform that demonstrated the synergy of experiences for both Māori and non-Māori, community and professional, Tangata Whenua and Tangata Tiriti, while also accommodating the unique individual experiences of these two distinct groups. These mechanisms enabled power sharing, examination of relationships with Māori, normalisation of Māori worldviews, and a focus on consistent practice, which are a must for equity and a continual commitment to Te Tiriti (Kidd et al. 2022).

Within wānanga, the HMC and HHcC acknowledged that te reo assessments are urgently needed, especially with the growth of te reo proficiency and support in Aotearoa (Stats 2022). With the development of te reo assessments underway, several challenges of implementing te reo and providing culturally appropriate and responsive care were raised. One significant challenge among HHcC was the ‘fear of getting it wrong’. Both communities resoundingly saw the potential for positive outcomes in addressing these fears as a profession. The HMC suggested manaaki (ethic of respect and care), aroha (compassion), and mana-enhancing (self-esteem enhancing, mutual respect and commitment to caring for each other’s mana) learning as ways to awhi (support) healthcare professionals’ journeys. These responses were firmly grounded in qualities highly prized in their Māori worldview.

In contrast, HHcC discussions centred on the need for organisational support in providing culturally responsive care and the use of indigenous frameworks and concepts, such as tuakana-teina model type learning (scaffolded modelling). Several education templates exist, with the HHcC mentioning the likes of cultural safety training, and the ‘Hui Process’ and ‘Meihana model’ within clinical assessments; a significant addition to how training and practice can be responsive to the diverse needs of Māori (Pitama et al. 2014). These models are yet to be implemented within Aotearoa hearing healthcare education, which is consistent with the limited culturally responsive ear and hearing health training education and research in other high-income colonial-settler nations (Nash et al. 2023). Improvements in the ear and hearing healthcare professional development infrastructure are therefore needed, so professionals can gain further skills, knowledge, and confidence in working with Māori and their whānau.

Many research partners were realistic that individual professional development could only contribute so much before there would need to be sector-wide systemic change. Both communities noted multiple barriers preventing Māori from obtaining quality hearing healthcare and optimal ear and hearing health outcomes. For example, lack of accessible information on existing services and funding, racism and discrimination, limited time with health professionals, and poor communication between services. Along with HMC and HHcC whakaaro, experiences of middle ear conditions, hearing loss and hearing healthcare among Māori, as well as aspirations for equitable systems and outcomes are documented in other Māori-led studies (Crisp 2010; Smiler 2014; Manuel 2022; Lowe 2023; Wai Rangahau 2023; Buckthought et al. 2024). These authors highlighted the long-standing impacts of colonisation on Māori with experiences of hearing loss/middle ear disease, emphasising challenges in accessing te reo, te Ao Māori, healthcare, employment and other social determinants of health. Similarly, this study emphasised the significance of Te Tiriti partnerships, whānau engagement and whānau-centred holistic care, Māori workforce, tikanga and mātauranga Māori, cultural safety and cultural support to achieve equitable ear and hearing healthcare and outcomes.

Tiriti-compliant partnerships recognise tino rangatiratanga adequately, including active protection of Māori rights to autonomy, self-governance, and to manage all aspects of Māori affairs in accordance with their own tikanga (Waitangi Tribunal 2019). Such partnership would therefore require the hearing health and education sectors to stay well-informed of relevant circumstances, and, if need be, provide additional resources to address the causes of inequities. HMC and HHcC agree that any transformation of the scope of provided support and services should be responsive to diverse Māori realities. Accordingly, grassroot efforts are needed to advance collective Māori determination and autonomy (Reweti 2023). This will facilitate the realisation of challenges, solutions, and wāwata of hearing, hard-of-hearing and d/Deaf Tangata Whenua to articulate a vision that is, in the words of Ngāi Tahu Rangatira, Tā Tipene O’Regan (2014), ‘shaped and coloured by us’. (p. 16)

Further work is required to develop te reo hearing assessments (e.g. validation of TRMDTT), and beyond that make systemic changes in ear and hearing health education and healthcare (e.g. community-based healthcare and equitable policies). Ongoing collaborations with research partners and kaupapa Māori research in the Master of Audiology programmes at the University of Canterbury and University of Auckland will assist in growing this work. In many ways, the wheel does not need to be reinvented with other whānau-centred Kaupapa Māori and te reo initiatives existing in Aotearoa that have and could meet whānau, hapū, and iwi hearing health needs. Tiriti-compliant partnerships will be fundamental to the development and action of a long-term strategy that will contribute towards the revitalisation of te reo and changes required to deliver culturally responsive, safe, and equitable hearing healthcare and outcomes in Aotearoa.

We conclude this wānanga article with a karakia whakamutunga (closing thought/prayer), drawing from the strengths and wāwata within this kaupapa to continue efforts in the revitalisation of te reo and equitable hearing healthcare and outcomes.

*Unuhia, unuhia. Unuhia ki te uru tapu nui.*

*Kia wātea, kia māmā, te ngākau, te tinana, te wairua i te ara takatā.*

*Koia rā e Rongo, whakairia ake ki runga.*

*Kia tina! Tina! Hui e! Tāiki e!*
